# Monitoring transformation of two tropical lignocellulosics and their lignins after residence in Benin soils

**DOI:** 10.1038/s41598-021-01091-y

**Published:** 2021-11-02

**Authors:** Rodrigue Daassi, Pierre Betu Kasangana, Damase P. Khasa, Tatjana Stevanovic

**Affiliations:** 1 Renewable Materials Research Centre (CRMR) and Institute of Nutrition and Functional Foods (INAF), Université Laval, Quebec, QC G1V 0A6 Canada; 2 Centre for Forest Research and Institute of Integrative and Systems Biology, Université Laval, Quebec, QC G1V 0A6 Canada; 3 Centre d’expertise et de recherche en écopédologie, Université d’Abomey-Calavi, Cotonou, Benin; 4 Energy Reduction in Mechanical Pulping Research Consortium, University of British Columbia, Vancouver, BC V6T 1Z4 Canada

**Keywords:** Biochemistry, Biotechnology, Chemical biology, Biogeochemistry, Environmental sciences

## Abstract

Thermally assisted Hydrolysis and Methylation (THM), and 2D-heteronuclear single quantum coherence nuclear magnetic resonance (2D HSQC NMR) spectroscopy were used to monitor the transformation of ramial chipped wood (RCW) from *Gmelina arborea* and *Sarcocephalus latifolius,* together with their organosolv lignins, following soil incubation in Benin (West Africa). Mesh litterbags containing RCW were buried in soils (10 cm depth) and were retrieved after 0, 6, 12 and 18 months of field incubation. Chemical analysis showed that total carbohydrate content decreased, while total lignin content increased as RCW decomposition progressed. Ash and mineral content of RCW increased significantly after 18 months of decomposition in soil. Significant N-enrichment of the RCW was determined following 18 months incubation in soils, reaching 2.6 and 1.9 times the initial N-content for *G. arborea* and *S. latifolius*. Results of THM showed that the S + G sum, corresponding to lignins, increased with RCW residence time in the soils, in contrast to the response of compounds derived from carbohydrates, the sum of which decreased. Remarkably, lignin interunit linkages, most notably β-O-4′ aryl ethers, β-β′ resinol, β-5′ phenylcoumaran and *p*-PCA *p*-coumarate, survived after 18 months in the soil, despite their gradual decrease over the duration of the experiment.

## Introduction

Since the 1990s, the application of lignocellulosic materials, such as ramial chipped wood (RCW), to soils is considered a useful amendment practice for soil restoration, and enhancement of organic carbon storage within soil ecosystems. RCW refers to the leafless branches (diameter < 7 cm) from trees and shrubs, preferentially from hardwoods, which are shredded and applied to soils by mulching or by direct incorporation into the uppermost 10 cm of mineral soils^1–3^. Like any wood materials, RCW is composed of structural constituents, such as carbohydrates (cellulose and hemicelluloses) and lignins, but they also contain large quantities of minerals and organic solvent-extractable molecules, in proportions that depend upon the type and origin of the materials^4–6^. Several studies have highlighted the positive effects of RCW used as a soil organic amendment. RCW amendments have been shown to improve physicochemical and microbial properties of soils^4,7,8^, by increasing the organic carbon and nitrogen content, which is promoted by soil biodiversity^7,9,10^. Further, they have been shown to enhance the phytoremediation abilities of soils^11–13^ and yields of several crops^1,6^. Cogliastro and coworkers examined the effect of application of woodchips from hardwood, by using litter bag system, and results indicated that it was beneficial for soil properties and crop production^14^. Moreover, Félix and collaborators indicated that RCW amendment mitigates degradation of tropical soils^6^. Beyond these beneficial effects that are accrued to crops, detailed characterization and transformation of structural components occurring within RCW in soils and, particularly that of lignins, has remained largely unexplored. The knowledge of the mechanism and the model of decomposition of the lignocellulosic material could allow a better understanding of the RCW biomass suitable for a good stabilization of carbon in soils. In this study, we monitored the degradation of RCWs of two tree species in the tropics, focusing on the distribution of their elemental composition, mineral content, and structural wood constituents, particularly lignins, as the RCW degradation proceeded in the soil.

Lignins are regarded as the major source of stable soil carbon, contributing about 20% to the total pool. They also contribute to carbon stabilization and turnover^15–18^, due to their relative recalcitrance, which is related to their aromatic structures and heterogeneity of intramolecular bonds^19,20^. In soils, the fate of lignins, along with lignocellulosic materials, has frequently been studied using copper oxide oxidation under alkaline conditions (CuO oxidation)^21–24^. Even though CuO oxidation has provided valuable quantitative data on lignin degradation in soils, the method has been largely supplanted by faster and more informative techniques, such as analytical pyrolysis coupled with gas chromatography and mass spectroscopy (Py-GC/MS)^25–29^. Py-GC/MS is a preferred technique, given that it permits rapid analysis of small samples while providing information that enhanced the insight into lignin transformations in soils^29^. Further refinement of the technique has been achieved through the introduction of tetramethylammonium hydroxide (TMAH)-assisted pyrolysis known as Thermally assisted Hydrolysis and Methylation (THM)^30^, which has proven to be useful for studying lignins and various other lignocellulosic materials in soils^31,32^. The originality of our approach resides in a parallel study of the transformation of the whole RCW following the soil residence, which has been recovered without soil contamination, which is a different approach from previous published data. The extraction of high purity organsolv lignins, at the prescribed time intervals, was developed in our laboratory and independently studied by spectroscopic and chromatographic analyses^2,33^. The high purity organosolv lignins are exempt from residual carbohydrates and ashes and therefore are adequate for structural studies. In particular, we have verified the hypothesis that the major substructure in all lignins, notably the β-O-4′ substructure, survives and to which extent during the decomposition in soil under the experimental conditions of this study.

The present study was designed to monitor the transformation of ramial chipped wood that was prepared from *Gmelina arborea* Roxburgh (gmelina, candahar or gamhar) and *Sarcocephalus latifolius* (JE Sm.) E.A. Bruce (African peach), and applied to soil at two sites in Benin (West Africa). The monitoring transformation was conducted at after 6, 12 and 18 months of soil incubation, using THM and 2D HSQC NMR spectroscopy techniques.

## Results and discussion

### Chemical composition of the *G. arborea* and *S. latifolius* RCWs after 0 (RCW0), 6 (RCW6), 12 (RCW12) and 18 (RCW18) months of soil incubation

**Figure 1:**
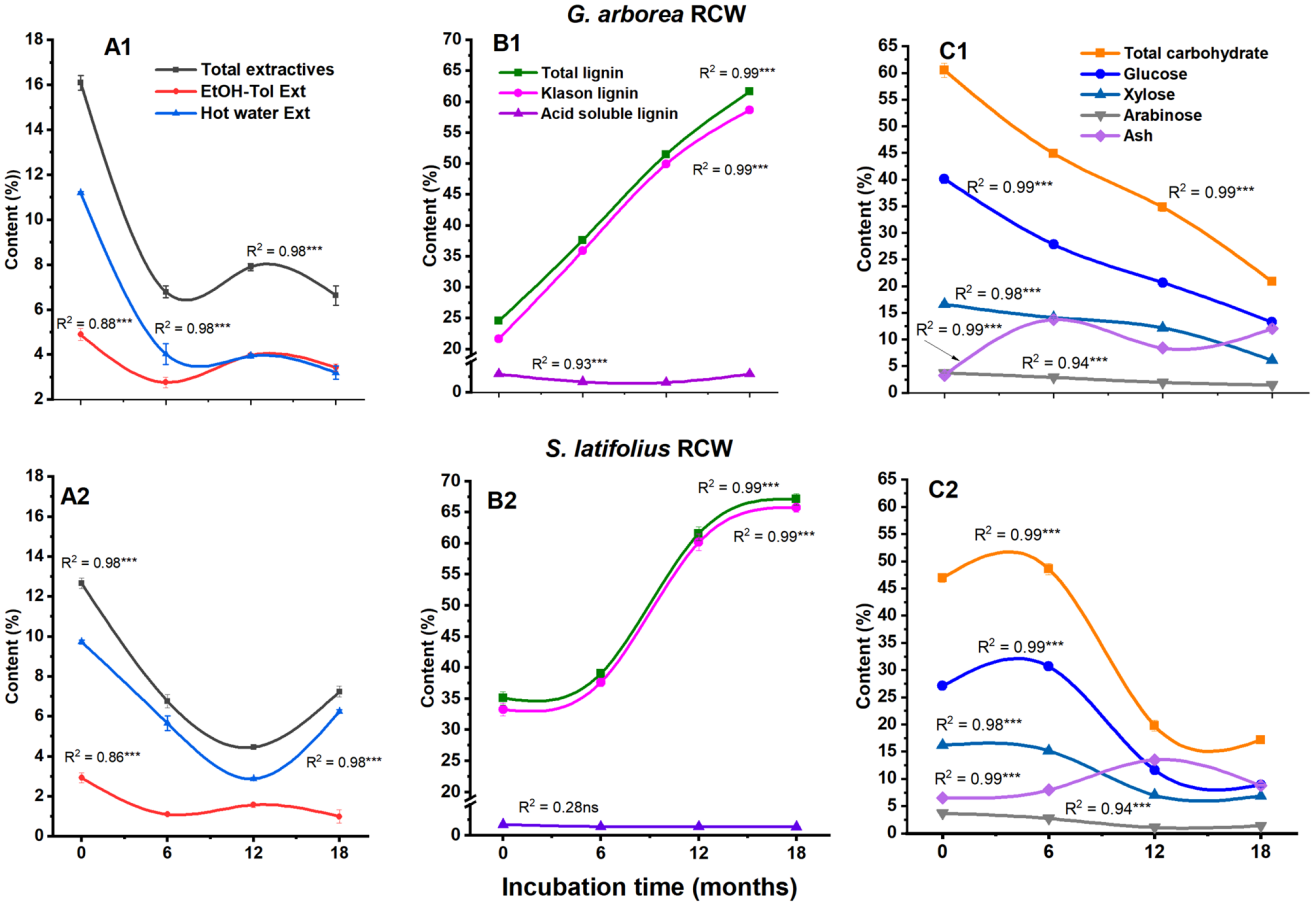
Content of chemical constituents of RCW of *G. arborea* (**A1**,**B1**,**C1**) and *S. latifolius* (**A2**,**B2**,**C2**) after 0, 6, 12 and 18 months of decomposition in soils. EtOH-Tol Ext, Ethanol-toluene extractives; RCW, ramial chipped wood, All contents are expressed as means ± standard errors (error bars) in triplicate. ***Contrasts significant at the 0.001 level, ns = not significant.

As presented in Fig. 1, the chemical constituents of studied samples varied significantly as shown by polynomial contrasts (linear and quadratic) with the progress of RCW decomposition in soils for *G. arborea* and *S. latifolius*. Total extractive contents decreased by more than 60% (compared to the initial content) for the two species after 18 months of soil incubation. This result is consistent with those described in other published reports, which indicated that extractive contents progressively decreased during wood decomposition^34,35^. Extractives, given that they are “free molecules,” most notably phenolic products, are the first to be released during decomposition. In both species, total carbohydrate contents also were initially much higher than total lignin contents (Fig. 1), which is consistent with what has often been reported in the literature, including our own work^29,36–38^.

As RCW decomposition progressed in soils, total lignin contents increased linearly, whereas total carbohydrate contents decreased with the same trend. For *G. arborea*, total carbohydrate loss occurred rapidly during the first 6 months of decomposition, in contrast to *S. latifolius*, for which the greatest carbohydrate loss was determined after 12 months. During organic matter decomposition, rapid initial losses of matter often can be attributed to leaching^36,39^. However, the rapid loss of water-insoluble components, such as polymer carbohydrates (polysaccharides), during the first 6 to 12 months of decomposition, suggests more rapid microbial catabolism of carbohydrates from RCW. The latter phenomenon seems to be much more pronounced for *G. arborea* than in *S. latifolius*. In addition to this response, several studies have reported that carbohydrates are preferentially degraded by brown-rot fungi (e.g., *Gloeophyllum* spp.) with a concomitant increase in relative lignin content with wood mass loss^21,40–43^. The decline in carbohydrate content could also be attributed to slow chemical autohydrolysis during decomposition in the soils^43^. Indeed, lignin is known to be recalcitrant to degradation relative to other wood components. Only a limited range of microorganisms, primarily white-rot fungi (Basidiomycotina), are reported to be capable of extensively depolymerizing the lignin structures^21,41,44^.

Ash content of the RCW increased with linear (*p* < 0.0001) and quadratic (*p* < 0.0001) trends as the RCW decomposition proceeded, reaching up to four and two times the initial ash content of RCW of *G. arborea* and *S. latifolius*, respectively. As indicated in our previous work^2^, the initial ash contents of study RCW were comparable to those reported in the literature. The significant increase of ash contents after 18 months of RCW decomposition suggests the potential contribution of microbial activity to mineralization, leading to release of minerals favourable for soil fertility and crop nutrition.

### Remaining mass, elemental analysis and degradability indices of RCW of *G. arborea* and *S. latifolius* during soil incubation

**Figure 2:**
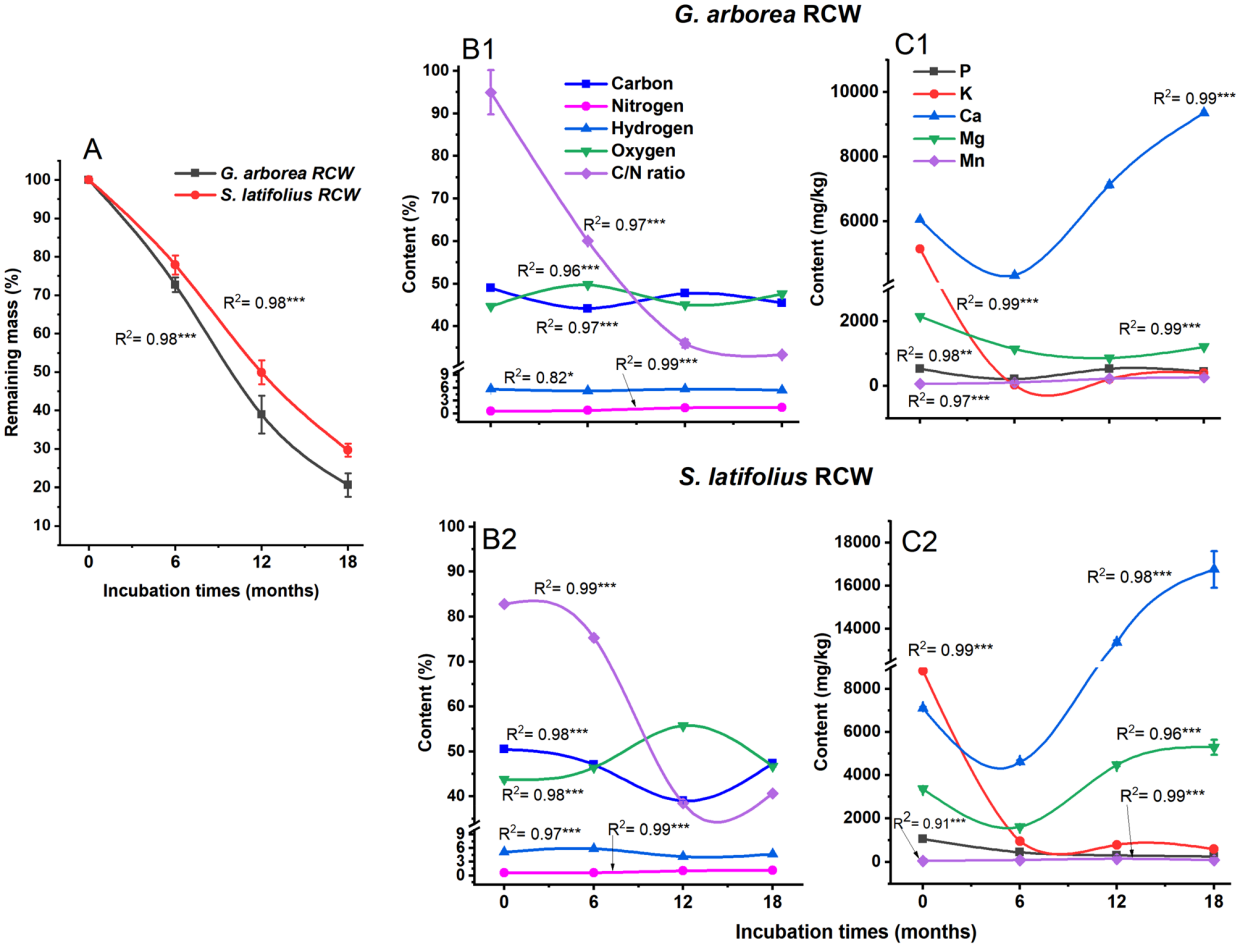
Content of chemical constituents of RCW of *G. arborea* (**A**,**B1**,**C1**) and *S. latifolius* (**A**,**B2**,**C2**) after 0, 6, 12 and 18 months of decomposition in soils. EtOH-Tol Ext, Ethanol-toluene extractives; RCW, ramial chipped wood, All contents are expressed as means ± standard errors (error bars) in triplicate. ***Contrasts significant at the 0.001 level, ns = not significant.

Figure 2 summarizes the data on remaining mass (Fig. 2A), elemental composition and degradability indices (Fig. 2B) and mineral content (Fig. 2C) of the study RCW. RCW mass losses decelerated over time for *S. latifolius* and *G. arborea* and had linear trend after 6, 12 and 18 months in the soils (Fig. 2A). The total mass loss of RCW throughout the decomposition period was higher for *G. arborea* (80%) than for *S. latifolius* (70%). The percentage of maximum mass loss was recorded between months 12 and 18 for *S. latifolius* RCW and between months 0 and 6 for *G. arborea* RCW. This difference in mass loss between the two species could be associated with the high amount of labile C in the carbohydrate and extractive components of *G. arborea* RCWand high quantities of recalcitrant C that was associated with the lignin of *S. latifolius* RCW, as reported in our previous work^2^. The C content for both species showed a relative decrease in time, while the O contents generally exhibited a slight increase, which was probably related to oxidative microbial transformation.

More interestingly, the N content of RCW increased substantially, reaching 2.6 and 1.9 times the initial content for *G. arborea* and *S. latifolius,* respectively. Progressive N-enrichment of RCW indicates that its application could improve the release and availability of N in soils, which is fundamental to maintaining soil fertility and important for plant nutrition. These results are in agreement with published reports, which also confirmed an increase of N content in decomposing biomass in soils^29,45,46^. This could be explained, at least in part, by an immobilization stage involving microorganisms, the enzymes that they secrete, and the protein cores of the former which contain nitrogen^45^. Pei and coworkers demonstrated that N released during decomposition can be immobilized by microorganisms, stabilized in the soil organo-mineral particles or absorbed to sustain crop productivity^38,46,47^.

C/N ratios are often used as degradability indices to explain the decomposition patterns of lignocellulosic materials under study. The initial RCW had a C/N ratio between 82.76:1 and 94.92:1 and, theoretically, would result in strong nitrogen immobilization. The C/N ratio of RCW progressively decreased throughout the 18 months of the study, achieving a value of almost one-third (around 33.32 ± 0.2) and half (40.65 ± 0.23) of the initial ratios that have determined respectively for *G. arborea* and *S. latifolius* RCW. There is a positive correlation (0.94, *P* < 0.001) between C/N ratio and the remaining mass; the C/N ratio of RCW at 18 months is slightly higher than 25:1, indicating N-immobilization, but to a lesser degree than in the initial stages of decay. For both species, there was a negative correlation between C/N ratio and N content (− 0.95, *P* < 0.001), indicating that C/N ratio was a key factor affecting N enrichment as reported by Pei et al.^46^.

Mineral contents of RCW (Fig. 2C) varied in various linear and quadratic trends with the RCW decomposition progress in soils for both species. Phosphorus and potassium contents significantly decreased, while calcium and manganese contents increased in RCW after 18 months of decomposition in soils for both *G. arborea* and *S. latifolius* RCW. The increase in manganese content could be related to the activity of white rot fungi since their enzymatic system comprises normally manganese-dependent lignin peroxidase^41,48^. Magnesium content decreased with RCW decay in *G. arborea*, while the opposite result was observed for *S. latifolius*. This relative variation in mineral contents reflects different dynamics of their release during RCW mineralization in soils, which is intended to improve nutrient status of the soils.

### Analytical pyrolysis Py-GC/MS of the RCW after residence in soils

Thermally assisted Hydrolysis and Methylation was conducted to follow changes in the composition of RCW from *G. arborea* and *S. latifolius* as these materials decomposed in the soil. Pyrograms of RCW from *G. arborea* and *S. latifolius* are shown in Figure S1 (supporting data). The compounds that are reported in Table 1 revealed that the syringyl guaiacyl (S + G) sum corresponding to lignins increased, while the sum of the compounds corresponding to carbohydrate content decreased. Several papers revealed that pyrolysis technique is less effective in detecting carbohydrate units in respect to the lignin component^49,50^. This is probably the result of pyrolytic rearrangement of polysaccharides in complex matrices combined with the derivatizing agent (TMAH) which prevent chemical access and are believed to negatively interfere in the diagnostic release of carbohydrates derivatives and polysaccharides components. However, these trends of decreasing carbohydrate content are also indicated by CH/L ratio (carbohydrate/lignin ratio), confirming the results of global chemical analyses that are presented in Fig. 1. The main guaiacyl (G) moieties that were released by RCW samples from *G. arborea* have been identified as peaks **10R**, **16R** and **22R** (Table 1), while the main syringyl (S) moieties of the same samples were identified as peaks **25R** and **26R**. Results showed that *G. arborea* samples contain GS-type lignin derivatives, dominated by S units, as confirmed by their lignin analyses presented below and reported in our previous study^2^. The S/G ratio of *G. arborea* samples increased slightly with RCW decomposition in soils, except for RCW18. This result could be explained by the fact that lignins from *G. arborea* are S-dominant. However, the moieties consisted of S units that are known to cleave more rapidly. Due to the easier cleavage of the ether bond by which the S-units are preferentially linked, the S unit content remained high. The very high initial S/G in *G. arborea* samples could perhaps explain the slight increase of the S/G ratio in the RCW samples even after 18 months incubation in soils. As for the RCW samples from *S. latifolius*, the main G moieties were identified (Table 1) in addition to those that were identified in *G. arborea* samples. The major compounds based on S units are the same as those identified in *G. arborea* samples, but with a lower relative abundance. The relative abundance of lignin derivatives units (S, G) (Table 2) showed that RCW from *S. latifolius* has GS-type lignin that is dominated by G units. The trend is linear (*p* < 0.0001) and, there is an indication of decrease in the S/G ratio, indicating greater recalcitrance of G-lignin. The lower initial value of the S/G ratio in *S. latifolius* compared to *G. arborea* is consistent with the results of other analytical methods (higher Klason lignin + acid solubles, higher C content) as has been reported in previous studies^28,30,31,51–53^.

**Table 1:** Identity and relative molar abundances of the compounds released after THM from the RCW samples at different durations of soil incubation; values are averages of triplicate measurements.

No	RT	Compound name	MW	Origin	Relative abundance (%)							
					*G. arborea* RCW				*S. latifolius* RCW			
					0	6	12	18	0	6	12	18
1R	7.99	Propane, 1,2,3-trimethoxy	134	CH	11.6	4	0	4.2	0	0	13.3	1.5
2R	11.73	Benzeneethanamine	121	N	0	0	0	0.4	0	0		0.7
3R	15.12	2-Furancarboxylic acid, 3-methyl-, methyl ester	140	CH	0.6	0	0	0	0	0	0	0.1
5R	17.86	1,2-Dimethoxybenzene	138	G	1.5	0.2	0	0.2	0	0.3	0	0.5
7R	18.58	1,2-Dimethoxy-4-(1,2-dimethoxyethyl)benzene	226	G	3.1	0.4	0	0.5	1.1	1.3	2.9	0.6
8R	21.29	3,4-Dimethoxytoluene	152	G	0.7	1	0	0.7	3.2	0.9	18.4	4.9
10R	25.69	Benzene 4-ethenyl-1,2-dimethoxy	164	G	10.9	4.8	7	6.2	5.6	9	9.7	8.4
11R	26.53	2,3,4-Trimethyllevoglucosan	204	CH	4.6	0.8	0	0.6	1.3	0.3	0	0.6
12	27.03	5-Methyl-1,2,3-trimethoxybenzene	182	S	0.2	1.2	2.3	0.6	0	4.9	2.5	0.4
13R	27.68	3,4-Dimethoxystyrene	164	G	0	1.7	1.1	1.3	38.9	0.3	26.5	12.3
14R	29.36	cis 3-(3,4-Dimethoxyphenyl)-propenoic acid methyl ester	252	S	6.4	2.2	0.7	0.4	23.5	4	6.5	11.5
15R	29.77	2-Butenoic acid-4,4-dimethoxy-methyl ester	160	CH	0	0	0.6	0	1	0.8	0	0
16R	30.40	Methyl Isoeugenol	178	G	2.7	1.5	4.9	1.3	3.2	2.1	0	4.5
17R	30.91	*trans* 3-(3,4-Dimethoxyphenyl)-propenoic acid methyl ester	252	S	8	0	0	0	0	0	0	0
18R	32.62	3,4-Dimethoxybenzoic acid methyl ester	196	G	0	0.6	0.6	0.3	0	0.8	0	1.1
19R	33.61	3,4,5-Trimethoxybenzaldehyde	196	S	0	0	0.4	0.7	3.9	0.4	7.8	0
20R	33.79	1-(3,4-dimethoxyphenyl)-1,2,3-trimethoxy propane	181	G	1.9	0	0	0	0	0	0	0
21R	33.97	3,4-Dimethoxypropiophenone	166	G	9.7	20.2	17.1	20.2	4.7	23.4	1.9	25.5
22R	34.97	Cis-1,2,4-Trimethoxy-5-propenylbenzene	208	G	0	5	5.7	6.8	1.2	5.8	2.1	4.5
23R	36.12	3,4,5-Trimethoxybenzoic acid methyl ester	226	S	0	0.3	0.4	0.4	0	0.3	0	0
24R	36.75	Methyl eugenol	178	G	0.4	0	0.4	0	1.6	4.7	1.7	3.4
25R	38.14	3′,4′,5′-trimethoxyacetophenone	210	S	6.1	13.1	32.5	26.9	2.1	18.5	2.8	9
26R	38.15	l,2,3,4-Tetramethoxy-5-(2)-propenylbenzene	238	S	13.5	29.3	14.6	15.8	0	5.4	0	1.7
28R	38.58	3,4-Dimethoxybenzyl methyl ether	182	G	0.4	5.8	0.9	2	1.5	8.1	0	5.7
29R	41.00	1-(3,4,5-trimethoxyphenyl)-1,2,3-trimethoxy propane	211	S	1.6	4.5	5.3	6.8	0.9	6.1	0	1.2
30R	41.43	Hexadecanoic acid, methyl ester	270	FA	2.5	2.4	2.1	1.5	1.5	1.5	0	1.3
31R	43.75	Pentadecanoic acid, 14-methyl-, methyl ester	256	FA	1.7	0.7	1.2	1.5	3.3	1.1	0	0.5
32R	44.61	Tetracosanoic acid, methyl ester	382	FA	1.7	0	0.6	0	0.6	1	0	0.2
33R	46.71	Octadecanoic acid, methyl ester	298	FA	1	0.8	2.2	0.9	0	0	0	0
34R	48.43	Alpha-D-glucopyranoside, phenyl, 2,3,4,6-tetra O-Methyl	312	CH	1.3	0	0	0	0.7	0	0	0

Each peak number is followed by R to refer to RCW.

RT, retention time (min).

**Table 2:** Lignin units (G and S), carbohydrate (CH), fatty acid (FA) and nitrogen containing coumpounds (N) molar contents from thermochemolysis using TMAH and SG sum, S/G and CH/L (L, lignin) ratios from thermochemolysis using of the RCW samples at different stages of residence in soils; the values presented are averages of triplicate measurements.

	S (%)	G (%)	CH (%)	FA (%)	N (%)	S + G sum	S/G	CH/L
***G. arborea* RCW**								
IT (months)								
0	35.73 ± 0.69	29.28 ± 1.43	17.97 ± 0.4	6.97 ± 0.78	1.86 ± 0.43	65.01 ± 21	1.22 ± 0.04	0.25 ± 0
6	50.5 ± 0.69	40.52 ± 1.27	4.81 ± 0	3.86 ± 0	0	91.02 ± 1.96	1.25 ± 0.02	0.05 ± 0
12	56.21 ± 1.1	37.11 ± 2.77	0.64 ± 0.13	6.03 ± 0.69	0	93.32 ± 3.87	1.53 ± 0.08	0.01 ± 0
18	51.48 ± 1.21	38.23 ± 0.92	4.76 ± 0.81	3.81 ± 0.52	0.44 ± 0.06	89.71 ± 2.13	1.35 ± 0	0.05 ± 0.01
Significance								
Linear	***	*	***	*	**	**	*	***
Quadratic	***	*	***	ns	***	**	ns	***
***S. latifolius* RCW**								
IT (months)								
0	30.48 ± 0.98	61.14 ± 1.1	3 ± 0.58	5.4 ± 0.64	0	91.62 ± 2.08	0.5 ± 0.01	0.03 ± 0.01
6	39.51 ± 1.27	55.89 ± 2.08	1.07 ± 0.06	3.53 ± 0.46	0	95.4 ± 3.35	0.71 ± 0	0.01 ± 0
12	19.57 ± 0.87	63.22 ± 0.69	13.32 ± 1.79	0	0	82.79 ± 1.56	0.31 ± 0.01	0.15 ± 0.02
18	23.76 ± 0.92	70.45 ± 2.6	2.22 ± 0.23	2.02 ± 0.35	0.73 ± 0.12	94.21 ± 3.52	0.34 ± 0	0.02 ± 0
Significance								
Linear	***	**	*	***	***	ns	***	*
Quadratic	*	**	**	**	***	ns	***	***

Results are expressed as means ± standard errors in triplicate; IT, RCW Incubation times (months) in soil, *Contrasts significant at the 0.05 level, **Contrasts significant at the 0.01 level, ***Contrasts signifi cant at the 0.001 level, ns = not significant.

### Yield, purity, and elemental analysis of organosolv lignins extracted from RCW as degradation proceeded in soils

It has been demonstrated previously^2^ that the organosolv process is well-suited to get access to high purity lignins from the RCW of *G. arborea* and *S. latifolius*, thereby increasing their suitability for studies of chemical structures. Table 3 presents the properties and elemental analysis of organosolv lignins that were isolated from *G. arborea* and *S. latifolius* RCW*.* The yield of organosolv lignin from the *G. arborea* RCW was greater than 15% and did not vary substantially with the progress of RCW decomposition within the experimental timeframe. However, the yield of organosolv lignin from *S. latifolius* RCW samples decreased with RCW incubation time, as reported in Table 3. For both species, lignin recovery decreased with RCW decomposition and this decrease is much greater for lignin of *S. latifolius* than *G. arborea.* Thermally assisted hydrolysis and methylation (THM) determined that more condensed structures accumulated in RCW lignins with increasing duration of incubation (Table 4), which could explain the difficulty of extracting greater proportion of lignins by the organosolv process. Nevertheless, the selected organosolv process seems appropriate, given that it yielded, from both species, organosolv lignins with purities higher than 95% (sum of Klason plus acid soluble lignin of the isolated lignins) and, therefore, suitable for structural studies. The elemental composition of the isolated organosolv lignins is presented in Table 3, revealing significant variation in C, H, O, and N contents. The presence of N in the lignins under study could be due to the potential formation of protein-lignin complexes during the organosolv pulping process^54^. Yet, this could also be explained by nitrogen presence in the native lignins, as nitrogen content has been determined in organosolv lignins extracted following all litter bag retrievals (0, 6, 12, 18 months) (Table 3). These findings indicate that lignin-protein chemical bonds are strong and, therefore, difficult to remove through pulping^2,54^. It should be noted that the N content of the lignins significantly increased as RCW decomposition progressed in soils. This was demonstrated for all RCW samples (Fig. 2), indicating the potential reactions between lignins and proteins from enzymes or microorganisms as the likely sites of nitrogen accumulation upon RCW degradation in soils.

**Table 3:** Properties and elemental analysis of lignins isolated from *G. arborea* and *S. latifolius* RCW at 0, 6, 12 and 18 months of decomposition in soils.

	Lignin yield (%)	Lignin recovery (%)	TL (%)	KL (%)	ASL (%)	Glucose (%)	Ash (%)	C (%)	N (%)	H (%)	O (%)
**RCW lignin (*G. arborea)***											
IT (months)											
0	15.9 ± 0.4	77.4 ± 1	99.2 ± 0.3	95.2 ± 0.3	4 ± 0.3	1.2 ± 0	0.3 ± 0	65.86 ± 0.06	0.81 ± 0.01	5.85 ± 0.02	27.44 ± 0.06
6	17.3 ± 0.8	52.5 ± 0.4	99 ± 0.5	95.2 ± 0.4	3.8 ± 0.2	0.6 ± 0	0.3 ± 0	66.01 ± 0.15	0.75 ± 0.01	5.78 ± 0.22	27.42 ± 0.06
12	15.6 ± 0.4	38.3 ± 0.4	96.3 ± 0.6	93.5 ± 0.2	2.8 ± 0.5	2.6 ± 0.2	0.8 ± 0	64.68 ± 0.37	1.14 ± 0.05	6.15 ± 0.27	27.97 ± 0.67
18	15.4 ± 0.3	26.1 ± 0.2 d	97 ± 0.6	94.6 ± 0.5	2.4 ± 0.1	0.9 ± 0.1	0.4 ± 0.1	62.08 ± 0.61	1.21 ± 0	5.83 ± 0.01	30.81 ± 0.64
Significance											
Linear	ns	***	**	ns	**	ns	*	***	***	ns	***
Quadratic	ns	***	ns	ns	ns	**	*	**	*	ns	*
**RCW lignin (*S. latifolius)***											
IT (months)											
0	16.3 ± 0.4	60.4 ± 0.4	98.4 ± 0.8	93.2 ± 0.4	5.2 ± 0.6	1.2 ± 0.1	0.7 ± 0	66.38 ± 0.02	0.78 ± 0.01	5.76 ± 0.02	27.02 ± 0.02
6	17.5 ± 1.2	52.3 ± 0.2	98.8 ± 0.3	95.4 ± 0.1	3.4 ± 0.2	0.7 ± 0	0.4 ± 0.1	66.31 ± 0.1	0.71 ± 0.01	5.65 ± 0.21	27.3 ± 0.3
12	11.6 ± 0.5	18.5 ± 0.5	95.4 ± 0.2	93 ± 0	2.4 ± 0.2	3.3 ± 0.1	1 ± 0	64.58 ± 0.19	1.06 ± 0.04	6.12 ± 0.11	28.19 ± 0.11
18	11.6 ± 1.1	17.9 ± 1.3	96.6 ± 0.7	93.9 ± 0.5	2.6 ± 0.2	1.4 ± 0.1	0.5 ± 0	63.81 ± 0.09	0.91 ± 0.01	5.72 ± 0.05	29.49 ± 0.05
Significance											
Linear	***	***	**	ns	***	ns	ns	***	***	ns	***
Quadratic	ns	***	ns	ns	*	ns	*	*	ns	ns	*

Results are expressed as means ± standard errors in triplicate; IT, RCW Incubation times (months) in soil; TL, total lignin; KL, Klason lignin; ASL, acid soluble lignin; *Contrasts significant at the 0.05 level, **Contrasts significant at the 0.01 level, ***Contrasts signifi cant at the 0.001 level, ns = not significant.

**Table 4:** Lignin units (G, S, and H), carbohydrate (CH), and nitrogen containing compounds (N) molar contents from thermochemolysis using TMAH and S/G ratios from thermochemolysis using of lignin isolated from the RCW samples at different stages of residence in soils; the values presented are averages of triplicate measurements.

	S (%)	G (%)	H (%)	CH (%)	N (%)	S/G
***G. arborea* RCW**						
IT (months)						
0	74.25 ± 1.44	24.47 ± 1.16	0.62 ± 0.06	0.52 ± 0.04	5.18 ± 0.32	3.04 ± 0.08
6	74.58 ± 1.24	27.75 ± 1.35	0.43 ± 0.02	0	2.44 ± 0.31	2.69 ± 0.06
12	86.43 ± 2.24	16.1 ± 1.12	0	0	2.57 ± 0.39	5.41 ± 0.3
18	83.17 ± 3.44	20.3 ± 1.11	0	0	1.63 ± 0.75	4.12 ± 0.16
Significance						
Linear	***	**	***	*	***	***
Quadratic	ns	ns	*	*	*	*
***S. latifolius* RCW**						
IT (months)						
0	48.12 ± 1.34	48.43 ± 1.95	1.04 ± 0.06	0	7.62 ± 0.85	0.99 ± 0.01
6	51.12 ± 1.44	44.88 ± 1.35	0.89 ± 0.06	0	9.21 ± 0.55	1.14 ± 0
12	55.3 ± 1.40	43.8 ± 1.15	0.46 ± 0.06	0	5.69 ± 0.35	1.26 ± 0
18	61.09 ± 2.44	43.41 ± 1.75	0	0	0	1.41 ± 0
Significance						
Linear	***	*	***		***	***
Quadratic	ns	ns	*		***	ns

N, protein-derived; H, p-hydroxyphenyl-derived; G, guaiacyl-derived; S, syringyl-derived; S/G, S/G ratio; IT, RCW Incubation times (months) in soil; Results are expressed as means ± standard errors in triplicate;*Contrasts significant at the 0.05 level, **Contrasts significant at the 0.01 level, ***Contrasts signifi cant at the 0.001 level, ns = not significant.

### Py-GC/MS of RCW *G. arborea* and *S. latifolius* lignins

**Figure 3:**
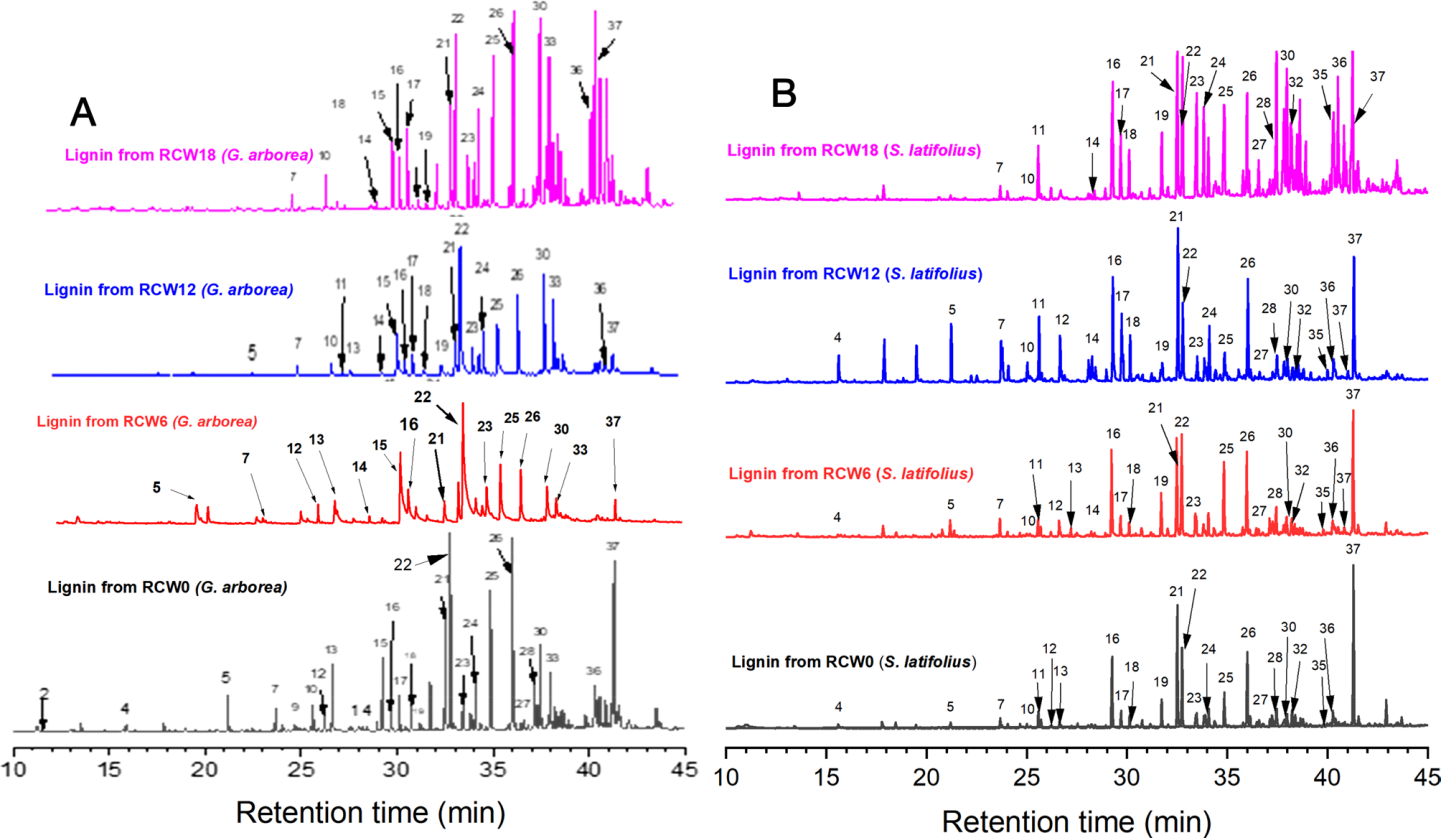
Pyrograms for RCW lignin from *G. arborea* (**A**) and *S. latifolius* (**B**). RCW 0: Initial RCW; RCW 6, RCW 12 and RCW 18 are RCW sampled after 0, 12 and 18 months in soils, respectively. Each peak number in this figure is followed by L to refer to lignin.

Organosolv lignins from *G. arborea* and *S. latifolius* were also analyzed by the Thermally assisted Hydrolysis and Methylation (THM), as was the case for the RCWs (Table 1). Figure 3 shows the chromatograms of pyrolytic products that were detected by THM for the four lignins of *G. arborea* and *S. latifolius*, respectively. The identified products and their relative abundances are listed in Table S1 (Supporting data). Based upon the figure and Table 4, the distribution of the pyrolytic products from the lignins showed linear and quadratic variation in their chemical structure as the RCW degradation progressed in soils. The main S-unit moieties that were present in the lignins were identified as peaks **15L, 16L, 18L, 22L, 25L, 26L, 30L, 37L** (L refers to lignin). While the G-unit moieties were identified as **7L, 10L, 11L, 17L, 19L, 21L, 23L,** and **35L**. A comparison of products from initial and decomposed RCW lignins of both species showed that benzene, cis-1-(3,4,5-trimethoxyphenyl)-1-methoxyprop-1-ene (peak 30) increased in abundance relative to other pyrolysis products upon degradation. The relative abundance of main compounds that were released and the S/G ratio of lignins are summarized in Table 4. S-unit abundance increased, while those of G-units decreased slightly for lignin samples as RCW decomposed in soils. *p*-hydroxyphenyl (H)-unit abundance is very low (0.33–0.94%), indicating that lignins are G-S type as determined previously. This distribution of lignin derivatives is similar to that observed following THM of lignin from several hardwoods^28,31^. Carbohydrate pyrolysis products such as anhydrosugars (1,6-anhydro-*β*-d-glucopyranose), pyrans and furans^28,52^ were not detected (Fig. 3), confirming the purity of the organosolv lignin. This reults also confirms the results regarding the high purity of the lignins under study (low carbohydrate content) (Table 3). The validity of choosing the organosolv process was affirmed, given that it led to high purity lignin preparations^33,55^. It shoud be noted that the recovery of lignin by organosolv process steadily decreased for lignins, which had been isolated from the RCW after 6, 12 and 18 months (Table 3). Nevertheless, the isolated lignins were determined to be of high purity, a compromise that must be made regardless of the method of lignin extraction that is chosen. The S/G ratio of the studied lignins steadily increased for the lignins isolated from RCW after long incubations in the soils, which could reflect the fact that a more easily soluble part of partially decomposed lignin has been recovered preferentially by the organosolv method that was applied with the same parameters throughout this study. This result could also indicate progressive depolymerization of lignin during decomposition, since it is well known that with increasing decomposition of woody biomass, the S/G ratio is usually increasing^15,21^.

### Polymer properties of lignins isolated from the RCW samples

Values of the number average (Mn), weight-average (Mw), and polydiversity index (PDI) are presented in Table 5. The molecular weight distribution curves are shown in Figure S2 (Supporting data). Mn values varied significantly with time. No significant difference for Mw and PDI was apparent over time or between species, but lignin that was isolated from RCW after 18 months of incubation in soil appears much less stable than that of the original material given that lower polydispersity is indicative of good physicochemical stability of lignin^56^. Mw and PDI both vary depending upon the lignin isolation process, molecular weight distribution methods, and the type of plant material and its degradation state^2,57^. In considering the Mw data of all samples as a function of their incubation time in soil, an increase (although not significant) was noted in the average molecular weight (Mw) of all lignin samples, from 1703 to 2028 g/mol for *G. arborea* and from 1692 to 2158 g/mol for *S. latifolius*. This response clearly indicates that lignin polymerization reactions with enzymes of microorganisms are taking place, as observed in the RCW analysis.

**Table 5:** Polymer properties of organosolv lignins isolated from the *G. arborea* and *S. latifolius* RCW at 0 (RCW0), 6 (RCW6), 12 (RCW12) and 18 (RCW18) months residence in soils.

	Mn (g/mol)	Mw (g/mol)	PDI
**RCW lignin (*G. arborea)***			
IT (months)			
0	652 ± 7	1703 ± 245	2.6 ± 0.4
6	468 ± 26	1457 ± 189	3.1 ± 0.6
12	552 ± 11	1929 ± 355	3.5 ± 0.7
18	678 ± 46	2028 ± 257	3 ± 0.2
Significance			
Linear	Ns	ns	ns
Quadratic	**	ns	ns
**RCW lignin (*S. latifolius)***			
IT (months)			
0	598 ± 18	1692 ± 328	2.8 ± 0.5
6	460 ± 9	1514 ± 218	3.3 ± 0.5
12	603 ± 29	2064 ± 304	3.5 ± 0.7
18	561 ± 29	2158 ± 324	3.9 ± 0.8
Significance			
Linear	Ns	ns	ns
Quadratic	Ns	ns	ns

Mn, number-average; Mw, weight-average; PDI, polydiversity index, results are expressed as mean ± standard error in triplicate; RCW Incubation times (months) in soil; Results are expressed as means ± standard errors in triplicate; **Contrasts significant at the 0.01 level, ns = not significant.

### 2D-HSQC NMR analysis of the organosolv lignins isolated from the RCW of *G. arborea* and *S. latifolius*

**Figure 4:**
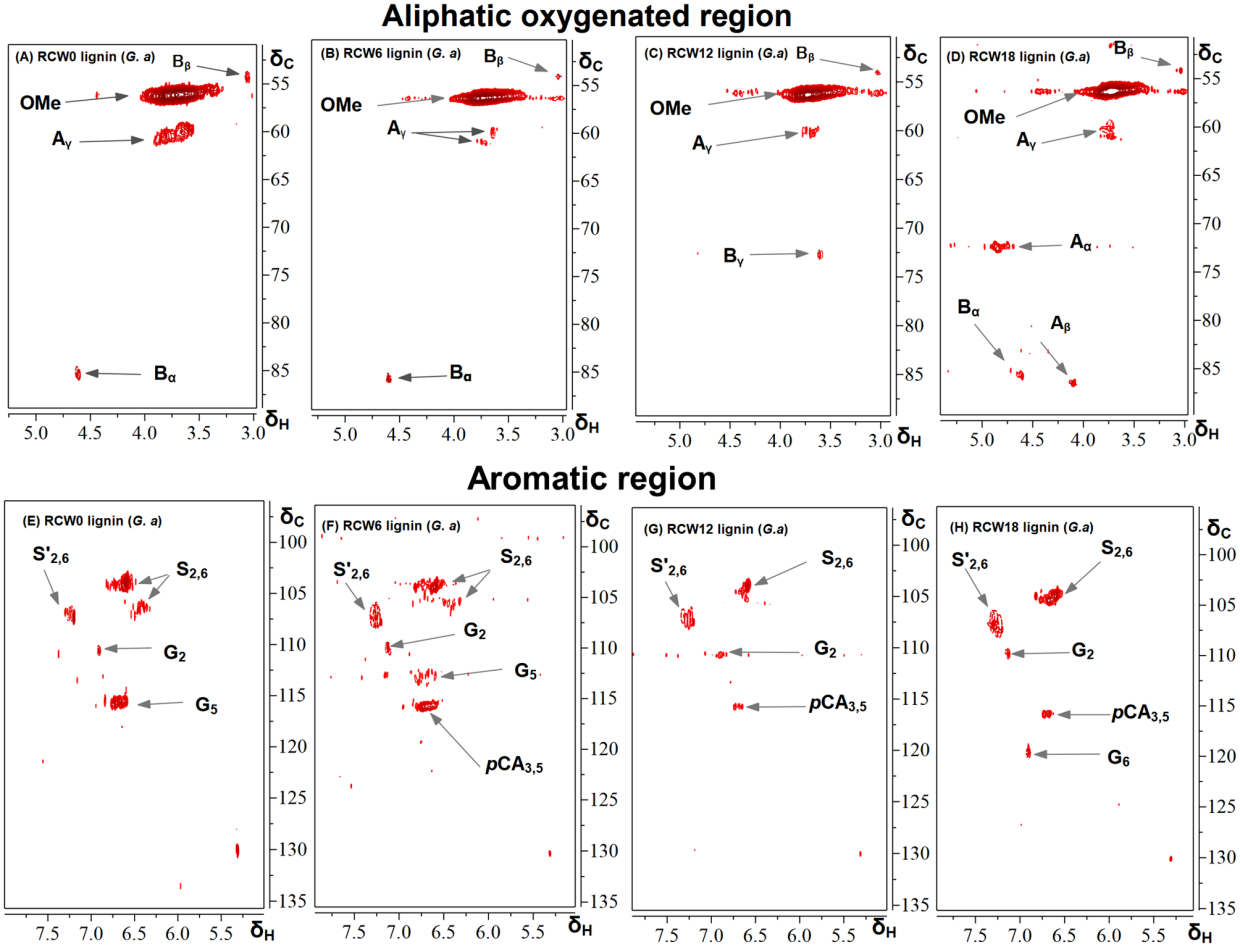
The 2D HSQC NMR spectra of RCW lignin from *G. arborea* (**A**–**H**). (**A**–**D**) Side chain (δC/δH 50–90/2.5–5.8) and (**E**–**H**) aromatic (δC/δH 90–120/5.5–8.0) regions in the 2D HSQC NMR spectra. G. a: *Gmelina arborea*, RCW0, RCW6, RCW12 and RCW18: RCW after 0, 6, 12 and 18 months of decomposition in soils, respectively.

**Figure 5:**
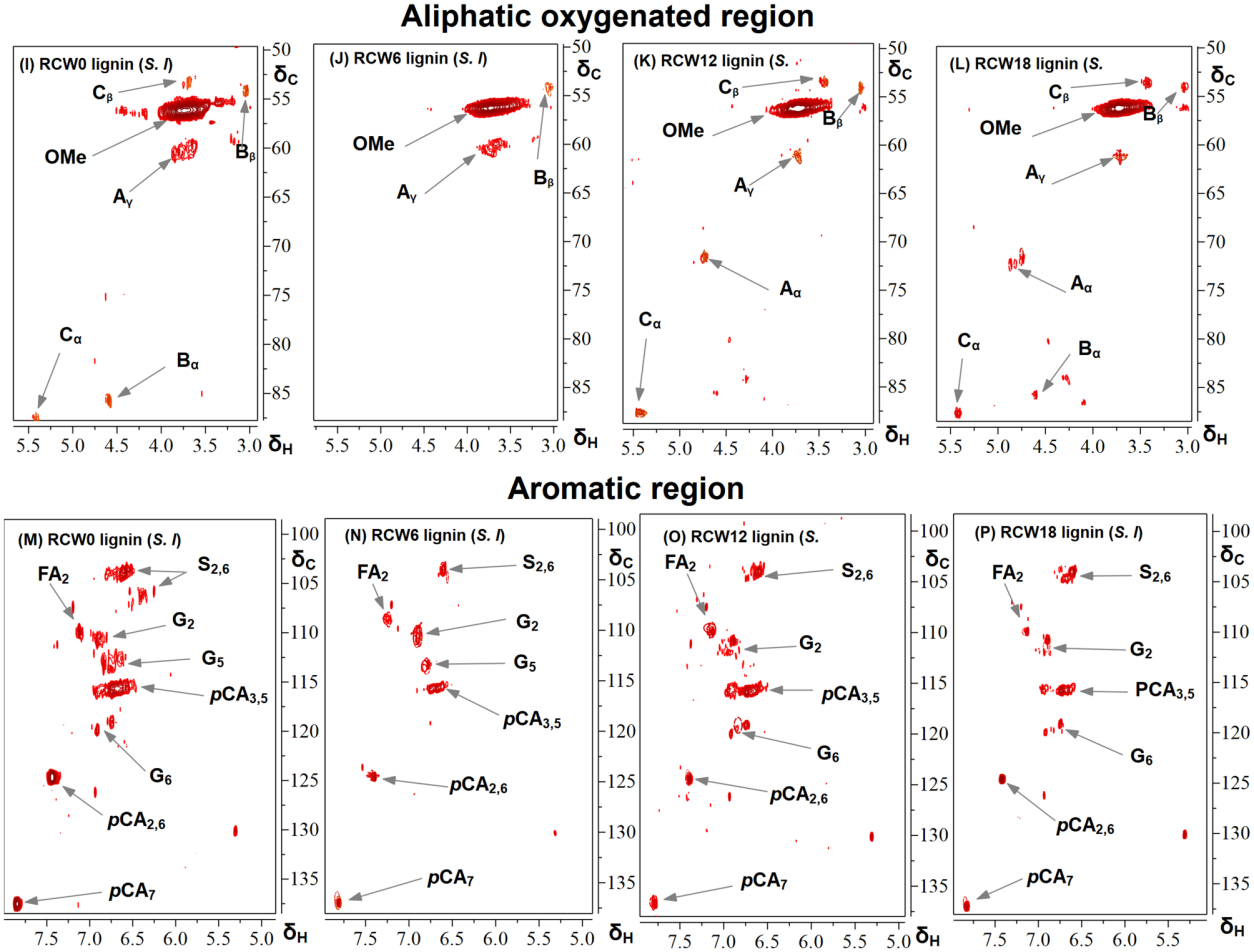
The 2D HSQC NMR spectra of RCW lignins from *S. latifolius* (**I**–**P**). (**I**–**L**) Side chain (δC/δH 50–90/2.5–5.8) and (**M**–**P**) aromatic (δC/δH 90–120/5.5–8.0) regions in the 2D HSQC NMR spectra. S. l: *Sarcocephalus latifolius*, RCW0, RCW6, RCW12 and RCW18: RCW after 0, 6, 12 and 18 months of decomposition in soils, respectively.

**Figure 6:**
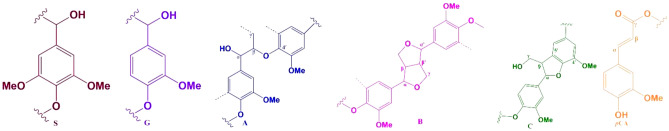
Main structures of organosolv lignins isolated from *G. arborea* and *S. latifolius* RCW, involving different side-chain linkages, and aromatic units identified by 2D HSQC NMR: (S) Syringyl unit, (G) guaiacyl unit, (**A**) β-O-4′ linkages, (**B**) resinol structures formed by β-β′, (**C**) phenylcoumaran structures formed by β-5′, and (*p*CA) p-coumarate.

To obtain additional information regarding the progress of changes in the studied lignin structures after incubation in the soil, 2D-HSQC NMR analyses were performed based upon methods that have been described in previous studies^20,57–59^. Figures 4 and 5 present the aliphatic oxygenated region (δ_C_/δ_H_ 50–90/2.5–5.8) and the aromatic/unsaturated regions (δ_C_/δ_H_ 90–155/6.0–8.0) of the 2D-HSQC NMR spectra of the organosolv lignins from the RCW of *G. arborea* and *S. latifolius*, respectively. The assignments of cross-signals that are related to the structural units and interunit bonds in the study lignins are listed in Table S.1 and are based upon data that have been taken from the literature^2,19,37,60^. The aliphatic oxygenated region of the spectra (Figs. 4, 5, top panel) have yielded useful information about the types of interunit linkages in the lignins under study. The correlation peaks (δ_C_/δ_H_) from methoxy (MeO) and β-O-4′ aryl ethers (A) were the most prominent in the HSQC spectra of the lignins of both species. It should be noted that this result confirms that the major interunit moiety of lignins does survive the decomposition process (Table 6). It is remarkable that the β-O-4′ moiety (Fig. 6), which could be regarded as the hallmark of lignin identity, is preserved. HSQC spectra revealed the presence of other characteristic signals which correspond to C_α_-H_α_ (71.8/4.83 ppm), C_β_-H_β_ (83.9/4.28 ppm), and C_γ_-H_γ_ (60.2/3.66 ppm) correlations for the β-O-4′ substructures (A), C_β_-H_β_ (86.9/4.08 ppm) correlations in γ-acylated β-O-4′ linkage. These β-O-4′ substrcutures were less prominent in the lignins from RCW corresponding to longer residence in soils. In addition, other correlation peaks are visible in the spectra including signals for β-β′ resinol (B) in all lignin samples and β-5′ phenylcoumaran (C), which is only present in the organosolv lignin from the RCW of *S. latifolius*. The presence of the latter confirms the importance of guaiacyl units for this lignin; this component has been determined for the lignin sample that was isolated from the initial RCW (time zero).

**Table 6:** Structural characteristics (lignin interunit linkages, cinnamyl end-groups, aromatic units and S/G ratio) obtained from integration of ^13^C–^1^H correlation signals in the HSQC Spectra of the lignin isolated from RCW of *G. arborea* and *S. latifolius.*

Characteristic	Lignin fractions							
	*Gmelina arborea*				*Sarcocephalus latifolius*			
	RCW0	RCW6	RCW12	RCW18	RCW0	RCW6	RCW12	RCW18
**Lignin interunit linkages (%)**								
β-O-4′ aryl ethers (A/A′)	63.34	50.14	42.4	37.18	65.67	52.79	39.57	34.92
Phenylcoumaran β-5 (C)					7.77		11.69	2.98
Resinols β-β′ (B)	9.83	4.02	2.79	2.51	20.1	10.78	13.28	7.49
***p*-hydroxycinnamates**								
*p*-coumarate substructures (*p*CA)		16.18	7.54	7.16		31.23	18.98	12.02
C7H7 in *p*-coumarate (*p*CA7)	–	–	–	–	21.13	22.67	18.59	12.22
C_2,6_-H_2,6_ in *p*-coumarate (*p*CA2,6)	–	–	–	-	17.99	15.92	12.57	11.25
**Lignin aromatic units**								
G	37.32	36.6	34.78	32.57	54.65	52.34	56.94	49.81
S	62.68	63.4	65.22	67.43	45.35	47.66	43.06	50.19
S/G	1.68	1.73	1.88	2.07	0.83	0.91	0.76	1.01

In the aromatic unsaturated region of the HSQC spectra (Figs. 4, 5, bottom panel), the main correlation peaks that were found correspond to the aromatic rings of different lignin units (S, G), and to *p*-hydroxycinnamates (*p*-coumarates, *p*CA). A strong characteristic signal of S and G units was identified by their correlation peaks at δ_C_/δ_H_ 104.2/6.61, 110.7/6.98, 115.5/6.67, 119.9/6.91 ppm corresponding to S_2,6_, G_2_, G_5_ and G_6_, respectively. Also, the signal corresponding to Cα-oxidized S-units (S′_2,6_) at δ_C_/δ_H_ 107.4/7.28 ppm was only observed in lignins from *G. arborea* samples. All study lignins were determined to contain a signal of *p*-coumarate (*p*CA), which was confirmed by important correlation (δ_C_/δ_H_) at 115.7/6.69 ppm (*p*CA_3,5_), while signals corresponding to *p*-coumarate in C_7_–H_7_ (*p*CA_7_) (137.3/7.87 ppm) and *p*CA2,6 (124.7/7.44 ppm) were only found in the spectral data for lignins from *S. latifolius*.

The relative content of the main lignin substructures, the molar content of S and G units, as well as the molar S/G ratio of the study lignins was evaluated from the contour integration volume in the 2D HSQC spectra. The results are presented in Table 6^20,57–59^. The semiquantitative results of the lignins under study demonstrate a predominance of β-O-4′ aryl ether linkages, which decreased from 63.34 to 37.18% for *G. arborea* and from 65.67 to 34.92% for *S. latifolius* as the duration of soil incubation increased. At the same time, the relative content of β-β′ linkages decreased from 9.83% to 2.51% in *G. arborea* and from 20.1% to 7.49% in *S. latifolius*, respectively. Furthermore, β-5′ substructure was only determined for *S. latifolius* samples and it decreased from 7.77 to 2.98% with increasing length of incubation.

The results indicate a progressive degradation of lignins with the increase in residence time of RCWs in soils, which seems to be more pronounced for lignin of *G. arborea* than that of *S. latifolius* (Table 6). Previous research has indicated that the cleavage of β-O-4′ linkages was the main mechanism of lignin depolymerization during wood decomposition^41,59^. This supports our result suggesting that RCW decomposition in soils is linked to the decline in β-O-4′ aryl ether linkages; nevertheless, they remain important moieties in the study lignins. Moreover, the S/G ratio of lignin is considered to be a major indicator of lignin depolymerization by white-rot fungi in soils^15,21,41^. The S/G ratios that were calculated increased from 1.68:1 to 2.07:1 and from 0.83:1 to 1.01:1 for RCW lignins that were isolated respectively from *G. arborea* and *S. latifolius*. Increased S/G ratios indicated that effective degradation of lignin was occurring during RCW decomposition, which agrees with thermochemolysis using tetramethylammonium hydroxide (TMAH) results. It is also important to note the incorporation of *p*-hydroxycinnamates in lignin of *Sarcocephalus latifolius* RCW, which agrees in part with results of the thermochemolysis using TMAH that are presented in Table 4.

## Conclusion

The study presented here provides insights into the transformation of the RCWs of *G. arborea* and *S. latifolius* and the fate of their lignins following incubation in the soil at two different sites in Benin. As RCW decomposition advanced, total carbohydrate and extractive contents decreased, while lignin content of RCW increased. The significant increase of ash contents of the RCW after 18 months of decomposition (up to 4 times) could suggest a potential contribution of microbial activity to mineralization, leading to release of minerals favourable for soil fertility and crop nutrition. The important enrichment in nitrogen of the RCW following 18 months incubation in soils, reaching 2.6 and 1.9 times the initial N- content for *G. arborea* and *S. latifolius*, respectively, could also be linked to microbial activity. This N- enrichment of the RCW indicates that their application could improve the release and availability of N in soils, which is fundamental to maintaining soil fertility and important for plant nutrition. Analytical pyrolysis showed an increase in the S + G products sum, which clearly indicated to the relative lignin enrichment of the RCW from *G. arborea* and *S. latifolius* after residence in soils. Furthermore, the S/G ratio of RCW from *G. arborea* increased slightly, while the opposite trend was observed for *S. latifolius* after long-term RCW decomposition. Organosolv lignins that were isolated from RCWs are G-S type and their purity was determined to be greater than 95%. An increase in S/G ratio was determined for isolated lignins by Py-GC–MS analysis, which was more prominent for *G. arborea* than for the *S. latifolius* lignin*.* This response indicated a pattern of slower decomposition in *S. latifolius* samples in soil compared to *G. arborea* samples, that could be favorable to soil organic carbon stabilization. Remarkably, the β-O-4′ substructures were the main substructures of all studied lignins; which remained to be important throughout the process of RCW decay. This is a significant finding, as it could indicate to the survival of the β-O-4′ substructures in lignins, after longer residence times in soils. These moieties were accompanied by β-β′, and *p*CA-containing moieties in both lignin samples, while β-5′ moieties were only detected in *S. latifolius* lignin, thereby confirming the great recalcitrance of these structures. The combination of analytical pyrolysis with spectroscopic and chromatographic methods that were applied in this study provided important insights regarding the progress of lignocellulosic decomposition and the fate of its lignins following incubation in tropical soils.

## Materials and methods

### RCW sampling and preparation

Ramial chipped wood (RCW) of *S. latifolius* and *G. arborea* was collected in May 2018, respectively from a 10-year-old plantation that was located near the Kika Agricultural Technical High School (09°17′09″N, 2°45′06″E; Northeastern Benin) and a 5-year-old plantation at the Mèdji Agricultural High School in Sékou (06°41′64″N, 02°34′82″E; Atlantic coastal Benin). Permission to the collection of young branches from both *S. latifolius* and *G. arborea* complies with institutional, national, and international guidelines and legislation (Certificate issued by the environmental NGO APRETECTRA, Cotonou, Benin). The selection of these wood species was based on their availability and proximity to the experimental sites applying the RCWs. RCWs were applied in an experimental design that is part of a larger agronomic trial at two sites (Sekou and Kika) with contrasting climate conditions in Benin. On one hand, Sekou site belongs to the Guinean region which is humid area with a bimodal rainfall patterm (average of 1200–1500 mm/year)^61^. Temperature and relative humidity ranged from 24.66 to 30.24 °C and 76.80 to 87.90%, respectively during the experimental period. Sekou soil is predominantly ferralitic (red soil) classified as Ferralsol (world reference base for Soil resources^62^) and Oxisol (US soil classification), and we have determined the following characteristics for the original soil: pH of 5.93, a C and N content of 7.41 g/kg and 0.77 g/kg respectively, a bulk density of 1.57 g/cm^3^ with a silty-sandy texture at the first 30 cm depth. On the other hand, Kika site belongs to the Sudano-Guinean agroecological area with a unimodal rainfall (average of 1100–1300 mm/year)^61^. The temperature ranged from 22.85 and 34.38 °C while relative humidity varied between 49.80 to 84.30% during the experimental period. Kika site is characterized by a ferruginous soil classified as Luvisol (world reference base for Soil resources^62^) and Alfisol (US soil classification) for which we have determined a pH of 6.6, C and N content of 10.72 g/kg and 0.97 g/kg respectively, bulk density of 1.24 g/cm^3^, and a silty-sandy texture at the first 30 cm depth. One should note that the site choice and consequently of their soils has been dictated by the larger framework of the project which involved the participation of two agricultural schools and their students in the project’s follow-up. For each species, 100 g of RCW (with 40.2% initial humidity) were placed in 20 × 20 cm nylon mesh litter bags (with 1 mm-mesh size, therefore porous and permitting contact with soil)^14,63^, which were incorporated into the soils at a depth between 1 and 10 cm, then the top of litter bags was levelled with soil surface. Sixty-four bags of each RCW species (with initial humidity determined at 40.2%) were initially buried in the soils in the experimental design. Buried RCWs are therefore in contact with the soil, which favored the decomposition process. We retrieved the nylon litter bags from the soils at six-month intervals for the total 18 month duration of the experiment. RCW samples were collected at four time periods: RCW0 (initial RCW sample not incorporated in soils); RCW6, RCW12, and RCW18 are RCW samples removed respectively after 6, 12 and, 18 months of residence in soils. At each sampling, the bags with RCW were carefully washed and rinsed in distilled water to separate them from the mineral soil particles as thoroughly as possible before oven-drying at 40 °C. The oven-dried RCW samples were weighed, then ground and sieved through a shaking screen. The sawdust fraction between 40 and 60 mesh was collected for chemical analyses.

### RCW chemical composition determination

Total extractive content of RCW samples was determined by Soxhlet extraction for 6 h with anhydrous toluene-ethanol (0.427:1.000, v/v), followed by hot water extraction. Klason and acid-soluble lignin contents of *G. arborea* and *S. latifolius* RCW were determined using standard methods^64^. Carbohydrates were determined using the protocol of National Renewable Energy Laboratory (NREL, Golden, CO, USA)^65^. The analysis was conducted by high performance liquid chromatography using a refractive index detector (HPLC-RID), and performed on an Agilent Technologies 1200 series system equipped with a Rezex RHM-Monosaccharide H + (8%) column (300 × 7.8 mm). Ash contents were determined according to the ASTM standard method^66^. Mineral contents (Ca, Mg, K, P, Mn) of study samples were determined using an optical emission spectrometer with excitation by inductively coupled plasma (ICP) on an Optimum Machine DV 4300 (Perkin Elmer, Norwalk, CT, USA) with a Scott-type of nebulizer. Elemental analysis of RCW samples were performed using a Perkin Elmer 2400 Series II (CHNO analyzer). The contents (%) of C, H and N in RCW sawdust are based on ash-free dry mass, while O content was computed by difference, i.e., O = 100 – (C + H + N). Each result corresponds to the mean of three replicates.

### Organosolv lignin extraction

The organosolv lignins were isolated from the RCW samples after 0, 6, 12 and 18 month soil incubations after pre-extraction of the biomass with ethanol–water mixture, followed by the pulping according to the procedure that was developed in our laboratory and demonstrated to provide high purity lignin (containing low residual sugars and minerals)^2^. Briefly, 100 g of extractive-free RCW biomass were treated with 1 L of ethanol–water mixture (1:1, v/v), and catalyzed with 6 mmol ferric chloride (FeCl_3_·6H_2_O) in a Parr reactor series 4842 (2 L) at 180 °C for 90 min. The residual liquor was obtained by vacuum filtration to separate it from the solid organosolv pulp, evaporated to remove ethanol, and then precipitated by acidification (2 M HCl, pH = 1.5). Finally, the organosolv lignin was recovered after filtration and oven-dried at 40 °C overnight. Lignin yield and recovery were calculated using Eqs. (1) and (2), respectively.

1 $$\text{Lignin yield} = \frac{\text{Isolated lignin mass }}{\text{Biomass weight}} \times {100 \%}$$

2 $$\text{Lignin recovery}=\frac{\text{Isolated lignin mass}}{{\text{Biomass weight}} \times {\text{initial Klason lignin}} }\times 100 \%$$

### Py-GC/MS analyses

RCW powder and its isolated lignins was submitted to Curie point pyrolysis in the presence of tetramethylammonium hydroxide (TMAH) as the derivatization agent for Py-GC/MS analyses. Analytical pyrolysis was performed using a filament pulse pyrolyzer (CDS Pyroprobe 2000, CDS Analytical, Oxford, PA, USA). The GC/MS system consisted of a gas chromatograph (Varian, CP 3800) and a mass spectrometer (Varian Saturn 2200, 30–650 u.m.a). We tested the pyrolysis of RCW samples without addition of TMAH, for which the relative abundance of detected peaks was very low. The addition of TMAH has therefore enhanced the methylation and thus overall volatility of pyrolysis products but also improved the extent of the pyrolysis process which yielded higher quantities of pyrolysis products available for detection. A 0.4 mg portion of the samples was impregnated with a 10 µL of TMAH in methanol at 25% prior to loading them into ferromagnetic tubes for drying for 30 s at 100 °C. The sample (RCW, lignin) was pyrolyzed according to the following program: both the GC injector and the temperature of the pyrolyzer transfer line were set at 250 °C and maintained for 10 s. The temperature of the transfer line was then increased to 550 °C at a rate of 20 °C/s and held for 10 s at the temperature. The carrier gas (helium) was operated at the flow rate of 1.0 mL/min. The mass spectrometer was set in electron impact mode (EI, 70 eV, m/z = 35–400) at 1 s per scan. Each chromatogram peak was identified with the mass spectral library of the instrument (NIST) and completed using data that were available from published work^28,30,31,51–53^. Three replicates were examined for each sample. Relative abundance of each compound was calculated by dividing its GC peak area by the summed areas of all selected pyrolysis products.

### 2D NMR analyses of lignins

About 80 mg of organosolv lignin sample was dissolved in 0.7 mL of dimethylsulfoxide-d_6_ (DMSO-d_6_); its nuclear magnetic resonance spectra were recorded on a Varian NMR spectrometer at 500 MHz at 25 °C in DMSO-d_6_ according to published methods^2,19,37,60^. The program used was the Bruker standard pulse program “hsqcetgpsi”. The following parameters were using per sample for a total data acquisition of 5 h: 2048 data points acquired from 10 to 0 ppm in F2 followed by a 1 s recycle delay, 160 to 0 ppm in F1 (13C) with 256 increments of 64 scans. Semi-quantitative analysis was performed using volume integration of contours in the 2D HSQC spectra to calculate relative numbers of inter-linkages and the syrinyl/guaiacyl (S/G) ratio of the study lignins.

### Molar mass distribution

The molar mass distribution of the study organosolv lignins was determined by gel permeation chromatography (GPC) on an Agilent 1200 series system under the following conditions: column, PL gel 5 μm Mixed-D 300 × 7.5 nm; mobile phase, tetrahydrofuran (THF); polystyrene, standard (580–28,770 Da, Agilent). Prior to injection, 40 mg of sample were dissolved in 4 mL of tetrahydrofuran and filtered through 0.45 μm porosity filter. The chromatographic analysis was performed in triplicate per sample. From the molar mass distribution, the number average molecular weight (Mn), the weight average molecular weight (Mw) and the polydispersity index (PDI = Mw/Mn) were determined.

### Statistical data analysis

Differences in chemical properties between RCW samples of study species that were recovered from soils, together with the polymer properties of isolated organosolv lignins, were performed using one-way analysis of variance. Orthogonal polynomial contrasts were used to determine regression polynomial significance (linear, quadratic) at 5%. All analyses were performed using the Statistical Analysis System (SAS) version 9.4^67^.

## Supplementary Information

Supplementary Information.
