# Supplementary material for: Monitoring transformation of two tropical lignocellulosics and their lignins after residence in Benin soils
Source: Sci Rep. 2021 Nov 2;11:21524. doi: 10.1038/s41598-021-01091-y (PMC8563747; doi:10.1038/s41598-021-01091-y)
Supplement: Supplementary file 1 — Supplementary Information. [file 41598_2021_1091_MOESM1_ESM.docx]

**Supporting data**

**Monitoring transformation of two tropical lignocellulosics and their lignins after residence in Benin soils**

Rodrigue Daassi, Pierre Betu Kasangana, Damase P. Khasa, and Tatjana Stevanovic

1. **Analytical pyrolysis Py-GC/MS of the RCW after residence in soils.**


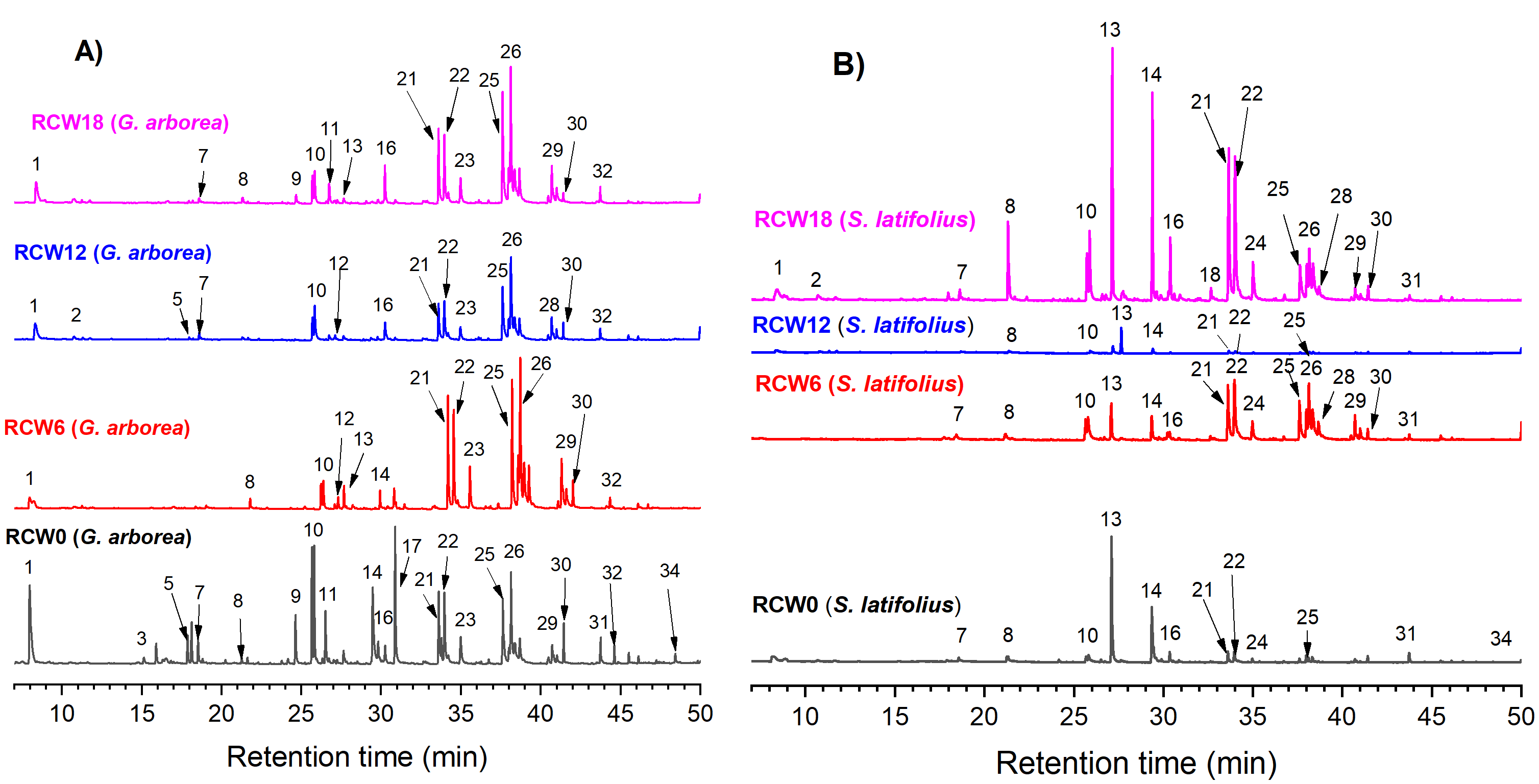


Figure S 1. Pyrograms of G. arborea (A) and S. latifolius (B) RCW.

*RCW 1: Initial RCW; RCW 2, RCW 3 and RCW 4 are RCW sampled after 6, 12 and 18 months in soils, respectively,* note that each peak number in Figure S 1 is followed by R to refer to RCW

## Py-GC/MS of the RCW *G. arborea* and *S. latifolius* lignins.

Table S1. Identity and relative molar abundances of the compounds released after TMAH-Py-GC/ MS of organosolv lignins that were isolated from RCW samples at different stages of decay in soils; the values presented are averages of triplicate measurements. Each peak number is followed by L to refer to lignins

| N° | RT | Compound name | Mw | Origin | Relative abundance (%) | | | | | | | |
| --- | --- | --- | --- | --- | --- | --- | --- | --- | --- | --- | --- | --- |
|  |  |  |  |  | RCW lignin (*Gmelina arborea*) | | | | RCW lignin (*Sarcocephalus latifolius*) | | | |
|  |  |  |  |  | 0 | 6 | 12 | 18 | 0 | 6 | 12 | 18 |
| 1L | 10.87 | Benzene, (methoxymethyl)- | 122 | H | 0.5 | 0.3 | - | - |  |  |  |  |
| 2L | 11.21 | Methyl, 2-furoate | 126 | CH | 0.5 | - | - | - |  |  |  |  |
| 3L | 11.62 | Methyl benzyl ether | 122 | H |  | - |  |  | 0.9 | 0.8 | 0.4 | - |
| 4L | 15.59 | 3,4-dimethoxyphenyl acetate | 196 | G | 0.9 | 1.6 | 0.3 | - | 1.4 | 1.6 | 5.3 | 0.7 |
| 5L | 21.18 | 3,4-dimethoxytoluene | 152 | G | 1.8 | 0.7 | 0.3 | - | 1.6 | 1.7 | 1.4 | - |
| 6L | 22.24 | 3,4-methyl-dimethoxybenzoate | 196 | G | - | - | - | - | 0.3 | 0.3 | 0.6 | - |
| 7L | 23.67 | Benzaldehyde, 3,4-di-methoxy | 166 | G | 1.8 | 1.6 | 0.9 | 0.7 | 3.7 | 3.2 | 7.8 | 0.8 |
| 8L | 24.67 | 2-methoxybenzhydrazide | 166 | N | - | - | - | - | 0.5 | 0.4 | 0.1 | - |
| 9L | 24.98 | (3,4-dimethoxyphenyl) dimethoxypropene isomer | 182 | G | 0.4 | 0.4 | - | - | 0.2 | 0.3 | 0.1 | - |
| 10L | 25.56 | 1,2-dimethoxy-4-vinylbenzene | 166 | G | 1.3 | 1.8 | 0.9 | 1.4 | 2.9 | 3.1 | 4.0 | 2.4 |
| 11L | 25.70 | 3,4-dimethoxybenzene acetic acid methyl ester | 210 | G | 0.6 | 1.0 | 0.4 | - | 4.5 | 5.5 | 12.7 | - |
| 12L | 26.21 | 4-methoxybenzhydrazide | 166 | N | 1.0 | 0.2 | - | - | 0.6 | 0.6 | 0.5 | - |
| 13L | 26.62 | 5-methyl-1,2,3-trimethoxybenzene | 182 | S | 4.4 | 1.7 | 0.9 | - | 0.6 | 0.5 | 0.2 | - |
| 14L | 28.38 | Methyl isoeugenol (E) | 178 | G | 1.6 | 4.3 | 1.9 | 2.1 | 0.5 | 0.6 | 1.9 | 2.6 |
| 15L | 29.23 | cis 3-(3,4-dimethoxyphenyl)-propenoic acid methyl ester | 252 | S | 4.5 | 9.5 | 5.3 | 3.5 | 0.3 | 0.3 | 0.7 | 0.5 |
| 16L | 30.11 | *trans* 3-(3,4-dimethoxyphenyl)-propenoic acid methyl ester | 252 | S | 4.4 | 4.6 | 4.0 | 3.1 | 3.9 | 3.6 | 5.8 | 1.9 |
| 17L | 30.73 | cis-2-(3,4-di-methoxyphenyl)-1-methoxyethylene | 194 | G | 1.7 | 1.1 | 0.8 | - | 9.1 | 9.1 | 7.7 | 6.5 |
| 18L | 31.19 | 1,2,3-trimethoxy-5-(2-propenyl)-benzene | 208 | S | 4.5 | 8.3 | 9.5 | 3.7 | 2.4 | 2.4 | 3.7 | - |
| 19L | 31.71 | 3,4-dimethoxyacetophenone | 180 | G | 3.1 | 3.3 | 1.5 | 6.9 | 3.6 | 4.4 | 2.2 | 3.3 |
| 20L | 32.03 | Acetamide, N-(3-ethylanmino)-4-methoxyphenyl | 165 | N | - | 0.4 | 0.3 | - | 0.5 | 0.9 | 0.5 | - |
| 21L | 32.49 | 3,4-dimethoxybenzoic acid methyl ester | 196 | G | 6.4 | 5.2 | 3.6 | 5.1 | 13.3 | 8.8 | 9.5 | 7.6 |
| 22L | 32.75 | 3,4,5-trimethoxybenzaldehyde | 196 | S | 11.8 | 16.7 | 16.8 | 10.0 | 9.1 | 9.7 | 5.1 | 6.7 |
| 23L | 33.42 | (3,4-dimethoxy-phenyl)-acetic acid methyl ester | 210 | G | 1.1 | 3.9 | 3.0 | - | 1.4 | 2.0 | 1.6 | 5.0 |
| 24L | 34.35 | 3,4-dimethoxypropiophenone | 194 | G | 1.1 | 0.2 | - | - | 1.2 | 1.1 | 1.1 | 2.4 |
| 25L | 34.85 | 3,4,5-trimethoxyacetophenone | 162 | S | 9.5 | 9.5 | 7.9 | 10.4 | 4.5 | 7.4 | 2.2 | 12.4 |
| 26L | 36.00 | 3,4,5-trimethoxybenzoic acid methyl ester | 226 | S | 12.5 | 6.4 | 9.3 | 10.0 | 9.7 | 8.8 | 8.1 | 7.7 |
| 27L | 36.59 | 3,4,5-trimethoxytoluene | 182 | S | 0.6 | 0.9 | 1.6 | - | 2.2 | 1.4 | 2.5 | - |
| 28L | 37.12 | threo-1-(3,4-dimethoxyphenyl)-1,2,3-trimethoxypropane | 270 | S | 2.5 | 0.1 | - | - | 0.8 | 1.5 | 0.2 | 0.9 |
| 29L | 37.28 | Ethanone, 1-(3,4,5-trimethoxyphenyl)- | 210 | S | 1.9 | 0.2 | 0.3 | - | 2.7 | 2.7 | 1.2 | - |
| 30L | 37.46 | cis-1-(3,4,5-trimethoxyphenyl)-1-methoxyprop-1-ene | 238 | S | - | 11.3 | 17.0 | 21.1 | 4.7 | 5.4 | 4.2 | 18.5 |
| 31L | 38.38 | Phenylpropylamine, N-acetyl-3,4-dimethoxy | 237 | N | - | - | - | - | 1.7 | 1.3 | 1.0 | - |
| 32L | 38.51 | 3',4',5'-trimethoxyacetophenone | 210 | S | - | - | - | 4.9 | 4.9 | 4.4 | 3.4 | 7.0 |
| 33L | 38.75 | cis-1-(3,4,5-trimethoxyphenyl)-1-methoxyprop-1-ene | 238 | S | - | 0.6 | 0.9 | - | 2.1 | 1.7 | 1.2 | - |
| 34L | 39.81 | 3,4,5-trimethoxyphenylethylamine | 211 | N | 1.6 | 0.5 | 0.6 | 1.0 | 0.8 | 1.2 | 0.7 | - |
| 35L | 39.96 | trans-1-(3,4-dimethoxyphenyl)-1-methoxy-1-propene | 208 | G | - | 0.6 | 0.6 | 2.2 | 2.5 | 1.4 | 1.6 | 10.3 |
| 36L | 40.15 | 1-(3,4,5-trimethoxyphenyl)-1-propene | 238 | S | 3.0 | 1.7 | 2.2 | 5.2 | 0.4 | 1.3 | 1.1 | 3.1 |
| 37L | 40.54 | cis-2-(3,4,5-trimethoxyphenyl)-1-methoxyethylene | 208 | S | 14.1 | 1.4 | 9.5 | 8.9 | 1.7 | 2.0 | 0.3 | 4.7 |

RT, retention time; S, Syringyl unit; G, guaiacyl unit; H, p-hydroxyphenyl unit; N, Nitrogen-based compound

1. **GPC results**


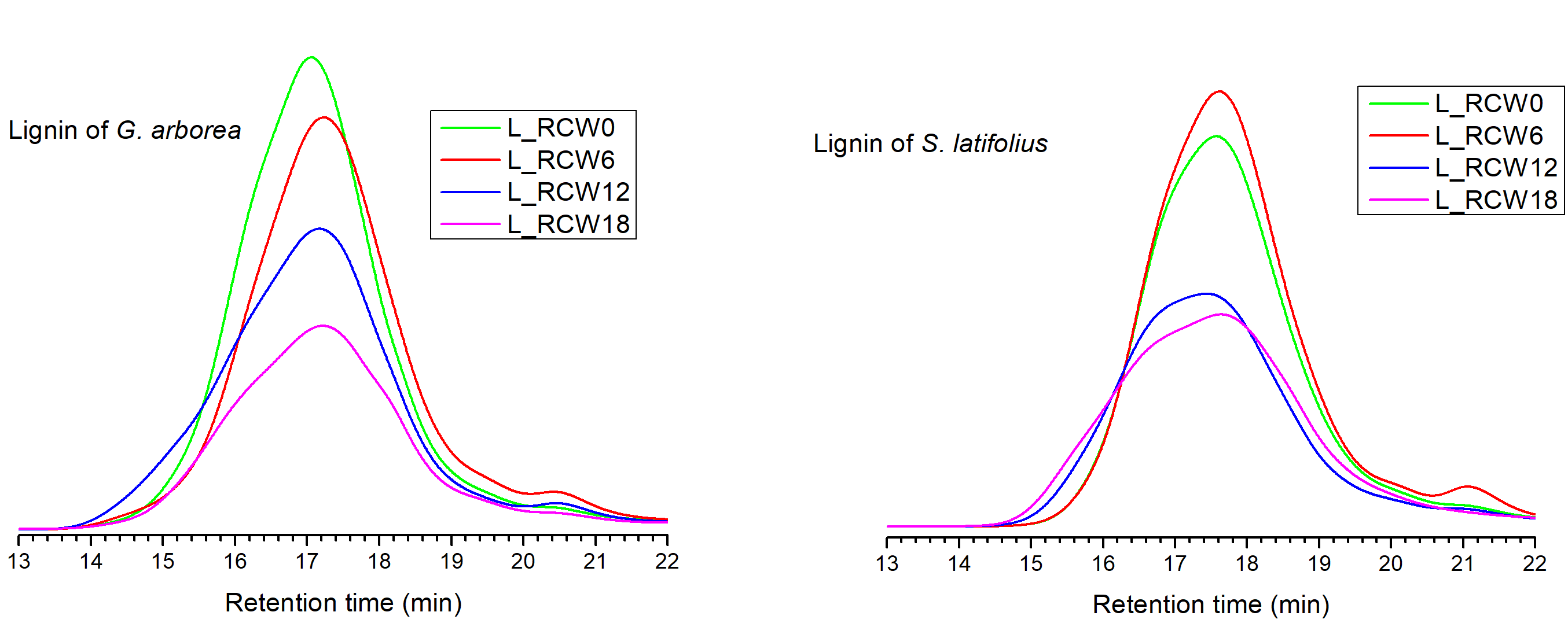


Figure S2. Molecular weight distribution of RCW lignins from G. arborea and S. latifolius. L_RCW0, L_RCW6, L_RCW12 and L_RCW18: lignin of RCW after 0, 6, 12 and 18 months of decomposition in soils

1. *2D* HSQC NMR analysis of RCW lignins from *G. arborea* and *S. latifolius*

Table S2. Assignments of the Lignin ^13^C−^1^H Correlation Signals in the 2D HSQC Spectra of lignins that were isolated from RCW of G. arborea and S. latifolius

|  | Label | δ_C_/δ_H_(ppm) | [Assignment](javascript:;) |
| --- | --- | --- | --- |
| 1 | Aα(S) | 71.8/4.83 | Cα−Hα in β-O-4′ substructures (A) linked to a S-unit |
| 2 | A_b_ (G) | 83.9/4.28 | C_b_-H_b_ in b*-O*-4 linked to G unit (A) |
| 3 | A_b_ (S) | 86.9/4.08 | Cβ−Hβ in γ-acylated β-O-4′ substructures linked to a S-unit (You et al. 2013; Popova et al. 2020) |
| 4 | A_b_ (S) | 72.5/4.86 | C_a_-H_a_ in b*-O*-4 linked to S-unit (A, thero) |
| 5 | B_a_ | 85.7/4.62 | C_a_-H_a_ in b-b resinol substructures |
| 6 | B_b_ | 54.0/3.04 | C_a_-H_a_ in b-b resinol substructures |
| 7 | A_g_ | 60.2/3.66 | C*_g_*-H*_g_* in b-*O*-4 substructures (A) |
| 8 | C_a_ | 87.5/5.42 | C_a_-H_a_ in phenylcoumaran substructures (C) |
| 9 | C_b_ | 53.5/3.45 | C_β_-H_β_ in phenylcoumaran substructures (C) |
| 10 | OMe | 56.2/3.71 | C-H in methoxyls |
| 11 | G_5_ | 114.9/6.72 and 6.94 | C_5_-H_5_ in guaiacyl units (G) |
| 12 | G_2_ | 110.7/6.98 | C_2_–H_2_ in guaiacyl units (G) |
| 13 | G_5_ | 115.5/6.67 | C_5_–H_5_ in guaiacyl units (G) |
| 14 | G_6_ | 119.9/6.91 | C_6_–H_6_ in guaiacyl units (G) |
| 15 | S_2,_**_6_** | 104.2/6.61 | C_2,6_-H_2,6_ in etherified syringyl units (S) |
| 16 | S′_2,6_ | 107.4/7.28 | C_2,6_-H_2,6_ in C*_a_*-oxidized (C_a_=O) phenolic, syringyl units (S’) |
| 17 | FA_2_ | 109.8/7.14 | C_2_-H_2_ in ferulate (FA) |
| 18 | *p*CA_3,5_ | 115.7/6.69 | C_3,5_-H_3,5_ in *p*-coumarate (*p*CA) |
| 19 | A_a_(S) | 71.5/4.73 | C_a_-H_a_ in b-*O*-4’ substructures linked to an S-unit (erythro) (A) |
| 20 | *PCE7 or p*CA_7_ and FA_7_ | 137.3/7.87 | C_7_-H_7_ in p-coumarate (PCE) |
| 21 | PCE2,6 | 124.7/7.44 | C_2,6_-H_2,6_ in p-coumarate (PCE) (Zhang et al. 2017) |
| 22 | CE-group | 126.1/6.93 | C_b_-H_b in C_innamaldehyde end-group |

1. Soil parameters at 18 months of incubation of RCW in soil

Table S3. Result of soil physical-chemical parameter at 18 months of incubation of RCW in soil

|  | pH | BD (g/cm3) | SOC (g/kg) | N (g/kg) | P (ppm) | C/N | CEC (meq/100g) | K (meq/100 g) | Ca (meq/100g) | Mg (meq/100g) |
| --- | --- | --- | --- | --- | --- | --- | --- | --- | --- | --- |
| RCW of *S. latifolius* | 6.1±0.23 | 1.23±0.06 | 15.93±1.58 | 1.13±0.22 | 23.65±2.8 | 14.01±2.26 | 25.8±3.86 | 1.16±0.37 | 4.28±0.23 | 1.28±0.07 |
| Control1 | 6.18±0.23 | 1.42±0.06 | 7.87±1.58 | 0.63±0.22 | 15.54±2.8 | 9.71±2.26 | 8.82±3.86 | 0.46±0.37 | 1.43±0.23 | 0.6±0.07 |
| RCW of *G. arborea* | 5.78±0.19 | 1.16±0.05 | 16.53±1.06 | 1.23±0.2 | 28.24±3.95 | 11.68±3.06 | 26.82±2.65 | 0.39±0.12 | 5.23±0.16 | 1.84±0.08 |
| Control2 | 5.55±0.19 | 1.26±0.05 | 6.37±1.06 | 0.47±0.2 | 9.23±3.95 . | 9.04±3.06 | 12.65±2.65 | 0.14±0.12 | 2.4±0.16 | 0.77±0.08 |

Control1: soil without RCW of S. latifolius; Control2: Soil without RCW of G. arborea; BD: bulk density; SOC: soil organic carbon content; N: nitrogen content; P: phosphorus; CEC: cationic exchange capacity Note that the Control1 and Control2 treatments correspond to the initial soil condition after 18 months of RCW incubation in the soil.
